# Comparative Micro-CT Analysis of Internal Adaptation and Closed Porosity of Conventional Layered and Thermoviscous Bulk-Fill Resin Composites Using Total-Etch or Universal Adhesives

**DOI:** 10.3390/polym17152049

**Published:** 2025-07-27

**Authors:** Dóra Jordáki, Virág Veress, Tamás Kiss, József Szalma, Márk Fráter, Edina Lempel

**Affiliations:** 1 Department of Restorative Dentistry and Periodontology, University of Pécs Medical School, Tüzér Street 1, 7623 Pécs, Hungary; dorajordaki@gmail.com (D.J.); veressvirag97@gmail.com (V.V.); 2 Department of Pharmacology and Pharmacotherapy, University of Pécs Medical School, Szigeti Street 12, 7624 Pécs, Hungary; kiss891012@gmail.com; 3 Department of Oral and Maxillofacial Surgery, University of Pécs Medical School, Tüzér Street 1, 7623 Pécs, Hungary; szalma.jozsef@pte.hu; 4 Department of Operative and Esthetic Dentistry, Faculty of Dentistry, University of Szeged, Tisza Lajos Blvd 64, 6720 Szeged, Hungary; meddentist.fm@gmail.com

**Keywords:** thermoviscous bulk-fill, layered resin composite, internal adaptation, adhesive strategy, micro-computed tomography

## Abstract

Reliable adaptation in Class II resin-based composite (RBC) restorations with margins on cementum remains challenging. This study compared the internal adaptation (IA) and closed porosity (CP) of three restorative strategies for such cavities, using either total-etch or self-etch adhesive approaches. Standardized box-only cavities were prepared on both proximal surfaces of 30 extracted molars, applying self-etch on mesial and total-etch on distal cavities. Group 1 used a layered microhybrid RBC; Group 2 used a flowable RBC base beneath a layered microhybrid RBC; and Group 3 used a thermoviscous RBC in a 4 mm bulk increment. Micro-computed tomography was employed to assess IA and CP. ANOVA, Tukey post hoc, and univariate analyses were used to evaluate group differences and the effects of adhesive/restorative strategies. Group 2 demonstrated the best adaptation (0.10%), whereas Group 3 exhibited the highest internal gap ratio (0.63%) and the lowest CP (*p* = 0.006). Total-etch adhesive significantly improved IA compared to self-etch (*p* < 0.001). These findings emphasize the impact of material selection and adhesive technique on the quality of restorations in cementum-located Class II cavities.

## 1. Introduction

The success of dental resin-based composites (RBCs) is contingent upon the internal adaptation of the material, which exerts a direct influence on the longevity of the restoration. Achieving an exact fit and minimizing interfacial gaps between the tooth structure and the restorative material is paramount in preventing microleakage and reducing the risk of postoperative sensitivity and secondary caries [1]. Minimizing interfacial gaps is also crucial in this regard. It is hypothesized that polymerization shrinkage of RBCs and the resultant shrinkage stress exert a deleterious effect on optimal internal adaptation by compromising adhesion to the tooth [2]. The extent of shrinkage is contingent on the composition of the RBC, primarily the quantity of filler, which determines the stiffness of the material during polymerization. However, the type of monomers utilized, the rate of the reaction, and the external constraints retrieved from the bonding to the tooth also have a significant impact [3,4,5,6,7]. Dental manufacturers have made efforts to address this deficiency. These endeavours have encompassed the formulation of advanced dental bonding agents and reduced shrinkage/shrinkage stress in RBCs. Furthermore, a range of clinical application methods have been employed to manage the shrinkage stress-induced complications. RBCs with a higher filler content, and thus higher elastic module, have become a widely adopted standard technique in the domain of layering placement [8]. Incremental application has been demonstrated to ensure adequate light cure penetration and to decrease shrinkage inherent to RBC by reducing the volume of cured layer [9,10,11].

The advent of single-increment bulk-fill RBCs, which can be utilized in thicknesses of up to 4–5 mm, was motivated by the objective of simplifying the filling process and eradicating the risk of air trapping [12]. Nevertheless, concerns have been raised regarding the shrinkage stress of bulk-fill RBCs as the material is applied in large volumes. In order to address this issue, a modified chemical composition with novel monomer technologies is employed in bulk-fill RBCs [13]. As demonstrated by comparative studies, it has been determined that there is no significant difference in the performance of bulk-fill and layered RBCs with regard to shrinkage stress and marginal adaptation. The preponderance of the findings from the experimental trials indicates a material-dependent nature of these parameters [14,15,16,17].

It was demonstrated that a negative correlation exists between the magnitude of shrinkage and shrinkage strain and the percentage of filler. It appears that this phenomenon may be attributable to a concomitant decrease in the volume fraction of monomers available for polymerization [18]. It is evident that the higher volume fraction of monomers plays a crucial role in the process of shrinkage increase. The lower elastic module of flowable RBCs allows the material to be applied as a stress-relieving thin layer under the RBC with a higher filler volume fraction [1,18]. A finite element analysis demonstrated that flowable RBC lining is capable of reducing polymerization shrinkage stress and occlusal force in enamel, dentin, the hybrid layer, and the adhesive layer to various degrees in tooth-restoration systems [19]. Furthermore, a reduction in RBC viscosity results in enhanced handling of the material and facilitates its application to cavities with complicated forms. This, in turn, leads to a reduction in the time taken for the procedure and an improvement in marginal adaptation [20].

However, should there be a wish to exploit the synergy of both parameters in conjunction with increased filler content and flowable consistency, RBC preheating constitutes a satisfactory alternative [21]. It has been demonstrated that the thermal energy transferred to the RBC has the capacity to enhance the molecular mobility of the resin monomers and the frequency of radical collisions. This enables the propagation process to persist for an extended duration prior to the initiation of autodeceleration [22]. The preheating of the RBCs has been demonstrated to enhance flow, reduce the film-forming thickness, and did not significantly affect other properties, such as linear shrinkage, flexural strength, and cytocompatibility [23,24,25]. Nonetheless, the extant literature on microleakage is the subject of considerable controversy. A number of studies have yielded favourable outcomes [26,27,28]. Conversely, other studies have not observed a decrease in microleakage. Nevertheless, even if microleakage is not improved, it is at least not adversely affected [29]. In contrast, other results demonstrated that the polymerization shrinkage strain of RBCs increased with elevated pre-polymerization temperatures [30]. In the absence of isothermal conditions, the abrupt decline in the temperature of the RBCs during the process of placement has the potential to interfere with the viscoelastic properties of the RBCs. This, in turn, may result in the material becoming detached from the walls prior to the onset of polymerization [23].

The problem has been overcome by the development of a solution that is specifically designed to preheat the material (VisCalor Bulk, Voco, Cuxhaven, Germany) in its delivery system (VisCalor Dispenser, Voco, Cuxhaven, Germany). This technology is known as Infrared Thermoviscous Technology. The solution in question allows a similar application to that of flowable RBC; however, due to the rapid cooling of the applied material, it can be sculpted immediately like a packable RBC, thereby reducing the gap created as a result of shrinkage after warming. The manufacturer asserts that the reduced volumetric shrinkage (1.44 vol%) and shrinkage stress (4.6 MPa) also play a significant role in preventing interfacial gap formation. The material under discussion offers a number of advantages. Firstly, it can be applied with no risk of void formation, and there is no requirement for an overlay. Furthermore, it exhibits excellent physical properties, which can be attributed to its high filler content (83 wt%). The validity of these claims has been substantiated by in vitro studies comparing VisCalor Bulk to regular bulk-filled RBCs [31], and moreover, the marginal integrity, which is critical to the success of RBC restorations, was found to be enhanced with VisCalor Bulk [32,33]. Conversely, the injection of the RBC into the cavity was found to be more significant for marginal adaptation than the preheating procedure [34]. In addition, preheated VisCalor Bulk, which is comparable to the other preheated bulk RBCs, exhibited a substantial increase in internal porosity when compared to the room temperature counterparts [35].

The decision-making process employed by dentists is not confined to the selection of RBC types; there is also the option to utilize different adhesive systems, such as total-etch, self-etch, or universal adhesives, when restoring teeth in disparate situations. An ex vivo study concluded that marginal integrity is not significantly influenced by the use of bulk-fill materials, bonding techniques, or variation in the location of cervical margins [17]. However, a subsequent systematic review determined that although the use of different adhesive protocols has an insignificant effect on the marginal adaptation and the bond strength of the interface between the material and the proximal dentin/cementum, the type of restorative material has a paramount effect on the test results [36].

The present study aims to evaluate and compare the internal adaptation (IA) and closed porosity (CP) across three distinct restorative strategies for Class II cavities located on cementum. The three strategies are as follows: conventional layered RBC, a combination of flowable base with conventional layered RBC, and preheated VisCalor Bulk, applying total-etch adhesive or a universal adhesive system in self-etch mode. This research utilizes a comprehensive analysis employing micro-computed tomography (micro-CT) to provide valuable data to the extant literature, thus guiding clinicians in their selection of materials for posterior restorations. The null-hypotheses were three-fold: (1) there is no difference in IA among the tested RBCs; (2) there is no difference in IA between the adhesive systems utilized, and (3) there is no difference in CP among the tested RBCs.

## 2. Materials and Methods

The study was approved by the Ethics Committee of the University of Szeged and the Medical Research Council of Hungary (BM/23566–1/2023) and adhered to the principles outlined in the Declaration of Helsinki. The restorations under study were made in extracted third molars that had been removed for orthodontic purposes. Informed consent was obtained from the patients. The extracted teeth were meticulously cleaned and stored in 0.9% saline solution for up to three months prior to use. To prevent microbial contamination, an antibiotic–antifungal mixture was used, consisting of 100 U/mL penicillin, 100 μg/mL streptomycin, and 2.5 μg/mL amphotericin B.

### 2.1. Specimen Preparation and Restorative Procedures

Table 1 presents an overview of the investigated materials, including their respective manufacturers and compositions.

The occlusal surface of the extracted teeth (n = 30) was reduced to a plane (919 diamond separator disc, Komet, Lemgo, Germany), 3 mm from the cemento-enamel junction, with the objective of standardising the size of the cavities and the uniform distance of the curing unit from the RBC.

Next, 3 × 3 mm wide box-only cavities were prepared (840G diamond cylinder bur, Hager & Meisinger, Neuss, Germany) with a vertical depth of 4 mm on the mesio- and disto-approximal surfaces of the teeth, according to the two types of adhesives utilised in the study. The gingival floor was set to terminate 1 mm apically from the cemento-enamel junction. The internal line angles were rounded. The oro-vestibular and mesio-distal dimensions of the cavities were measured using a digital caliper, with a precision of 0.01 mm (Mitutoyo Corp., Kawasaki, Japan). The 4 mm depth of the cavity along the axial walls was evaluated using a periodontal probe (546/1, Medesy, Maniago, Italy). Preparation of butt-joint cavosurface margins (~90°) was undertaken. The bur was replaced following the preparation of each tenth tooth.

Prior to the commencement of the restoration process, a Tofflemire universal matrix band retainer (Henry Schein, Melville, NY, USA) was securely affixed to the crown in conjunction with a contoured metal band (thickness: 0.04 mm, width: 6.3 mm; Polydentia, Mezzovico-Vira, Switzerland).

The mesial cavities were then subjected to a self-etch adhesive procedure in accordance with the manufacturer’s instructions. Prime & Bond Universal adhesive was meticulously rubbed onto the prepared cavity walls for 20 s, followed by a 10 s drying and 20 s polymerization period in standard mode (LED.D, Woodpecker, Guillin, China; irradiance: 1150 mW/cm^2^; wavelength: 420–480 nm; light guide tip diameter: 8 mm). The same curing unit was utilized for the entirety of the polymerization processes during the investigation. The tip of the fiberglass light guide was positioned at the centre of the sample, with a distance of 1 mm being maintained from the occlusal orifice of the cavities. Prior to each instance of polymerization, the light irradiance was measured and subsequently controlled using a radiometer (Bluephase Meter II, Ivoclar, Schaan, Liechtenstein).

The distal cavities were subjected to a total-etch adhesive procedure involving the application of 35% phosphoric acid (Ultra-Etch, Ultradent, South Jordan, UT, USA), initially on the enamel margins and subsequently on the dentin for a duration of 15 s. This was followed by a thorough washing process that lasted for 20 s. Thereafter, the cavity was gently dried. Subsequently, the Adper Single Bond 2 adhesive was applied, followed by a rubbing process for 10 s. This was then followed by a drying period of 10 s, and finally, a 20-second polymerization stage.

In Group 1, both the mesial and distal cavities were restored using Filtek Z250 microhybrid RBC in 1.5–2 mm oblique layers. Each layer was polymerized for a duration of 20 s.

In Group 2, Filtek Supreme Flow nanofill flowable RBC was applied in a 1 mm layer thickness to the gingival floor of both mesial and distal cavities. This was followed by the application of two subsequent ~1.5 mm thick oblique layers of Filtek Z250 microhybrid RBC. It is noteworthy that both the flowable RBC layer and the oblique condensable RBC layers were polymerized separately for 20 s.

The restoration of the mesial and distal cavities of Group 3 was achieved through the utilization of the thermoviscous VisCalor Bulk, which was applied in a 4 mm thick bulk increment. Prior to the application, the RBC was preheated in a gun-style variety VisCalor Dispenser using the T1 setting. The efficacy of this setting mode has been demonstrated through its ability to preheat both the warming device and the RBC to 68 °C within a span of 30 s.

The Filtek Z250 and VisCalor RBCs were subjected to a process of condensation, which was carried out at ambient temperature using a handheld instrument (LM-Arte Condensa, LM Dental, Parainen, Finland) that was operated manually.

The RBC restorations were finished and polished with a series of Sof-Lex discs (8691 C, M, F, SF; 3M, St. Paul, MN, USA). All the restorations were prepared by a single operator.

**Figure 1:**
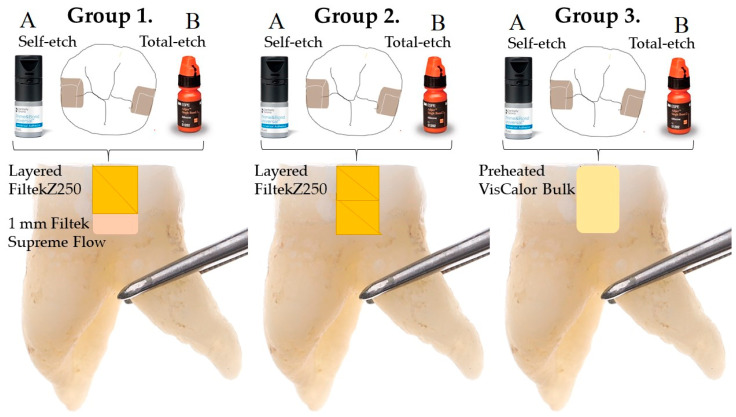
Schematic diagram of the tested settings.

The arrangement of the study groups is presented in Table 2. Figure 1 provides a visual representation of the restoration process applied to the investigated groups.

The restored teeth were stored in distilled water in an incubator (Cultura incubator, Ivoclar Vivadent, Schaan, Liechtenstein) at 37 °C for a period of three months, with the water being changed on a weekly basis. To prevent microbial contamination, an antibiotic–antifungal mixture was used, consisting of 100 U/mL penicillin, 100 μg/mL of streptomycin, and 2.5 μg/mL of amphotericin B.

### 2.2. Micro-Computed Tomography Measurement—3D Internal Adaptation and Porosity

**Figure 2:**
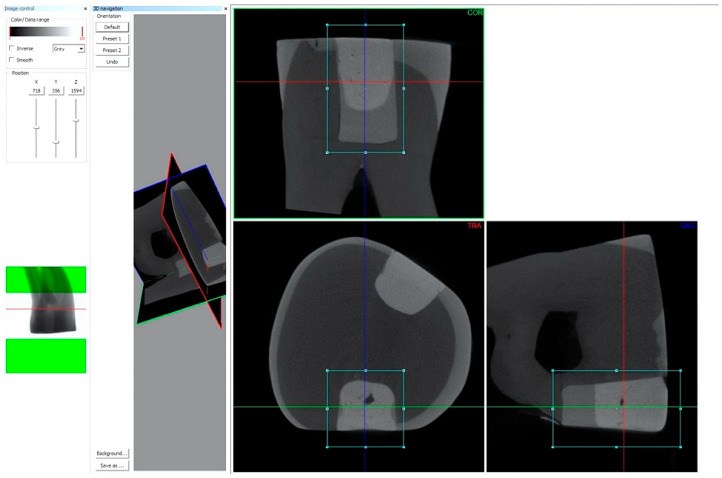
Reconstruction of the mesial and distal restorations in the tooth samples in coronal, axial, and sagittal views using the DataViewer software (Bruker, version 1.5.6.2).

The images were captured using a Skyscan 1176 micro-CT version 1.1 (build 12) (Bruker, Kontich, Belgium), with the samples positioned at the centre of the field of view, ensuring the axis of the recording field was parallel to the axial plane of the tooth samples. Each tooth was scanned for a duration of 35 min. The X-ray tube was operated with the following parameters during the measurements [37]: tube voltage: 80 kV, current: 310 µA, exposure time: 1500 ms, resolution (pixel size): 8.74 µm, filter: Al 1 mm. The reconstruction was performed using NRecon software (Bruker, version 1.7.4.2). The reconstructed image sequences contained two different fillings in the tooth samples. These were saved in separate image sequences in the axial, coronal, and sagittal planes using the DataViewer software version 1.5.6.2 (Bruker, Kontich, Belgium) (Figure 2). The raw images were subjected to a uniform reconstruction process, resulting in the creation of multiplanar image sequences. Subsequently, the images (initial pixel dimensions 8.74 µm) were converted to a full detector resolution of 1404 × 1404 pixels, employing the *.bmp format. The reconstructed image sequences were also analyzed using CTan software version 1.20.8.0+ (Bruker, Kontich, Belgium). The reconstruction process was executed in accordance with the following parameters: reconstruction duration: 0.8 s for each slice; reconstruction angular range: 197.4°; angular step: 0.7°; ring artefact correction set at 20; edge smoothing at 0.0; and the radius gain set at 20%. In order to achieve the maximum possible level of image detail, the filter cutoff relative to the Nyquist frequency was set at 100 (filter type: Hamming, Alpha = 0.54). A comprehensive dataset was utilized for the reconstruction process, with the undersampling factor fixed at 1, the threshold for the defect pixel mask set to 0%, and the beam hardening correction set at 20%. The reconstructed image sequences were also subjected to analysis using CTan software version 1.20.8.0+ (Bruker, Kontich, Belgium).

The solid volumes of tooth and RBC filling were determined according to the grey scale area. The grey scale in the ROI was representative of the solid volume of the samples, with dark colors denoting the gap volume between the filling and the corresponding tooth.

In order to analyze the 3D microarchitecture, the position of the reconstructed scan sequences was standardized in the coronal plane, so that the plane of the image slices was perpendicular to the vertical axis of the restoration (DataViewer: version 1.5.6.2 64-bit). The subsequent workflow was then applied to each tooth in order to evaluate the IA (interfacial gap) between the RBC restoration and the cavity walls. The procedure entailed the identification and manual delineation of the region of interest (ROI), including 0.1 mm (~10 voxel) tooth and 0.1 mm (~10 voxel) restoration along the tooth-restoration interface. Image filtering was employed for the purpose of noise reduction, thereby facilitating straightforward recognition of gaps at the interface by the software. Binary segmentation was utilized to enable uncomplicated separation of the object from the background. Gap identification was conducted in 3D using a binary mask derived from the lowest threshold range. The objective was accomplished through the implementation of a region-growing approach, whereby regions exhibiting equivalent density to that of air were selected. This process was initiated from a designated seed point and involved the incorporation of adjacent voxels that demonstrated analogous intensity. A 3D analysis was performed along the interface of the entire restoration (CTan software, version 1.20.8.0+). The ratio of gap volume to ROI volume was calculated and expressed as a percentage (internal adaptation in percentage, IA%).

In order to assess the CP volume, it was necessary to incorporate the entire RBC restoration within the ROI. The porosity was calculated using grayscale images that had been processed with a Gaussian low-pass filter in order to reduce noise. A histogram-based global thresholding method was applied to segment the grey scale images into binary images, assigning black and white values based on intensity ranges corresponding to material and void, respectively. The process of region growing was utilized for the isolation of internal voids. The CP volume relative to the total volume of the restorations was calculated as a percentage by measuring the internal voids and specimen volumes of each RBC sample.

### 2.3. Statistical Analysis

Sample size formula [38] and previous study results [37] were used to estimate sample size for micro-CT (IA% and CP) measurements.

$$Samplesizeformula:n=\frac{{\left(z_{1-\frac{\alpha }{2}}+z_{1-\beta }\right)}^{2}{\left(s_{1}+s_{2}\right)}^{2}}{(M_{1}-{M_{2})}^{2}}$$

where z = standard score; α = probability of Type I error at 95% confidence level = 0.05; z_1 − α/2_ = 1.96 for 95% confidence; β = probability of Type II error = 0.20; 1 − β = the power of the test = 0.80; z_1 − β_ = value of standard normal variate corresponding to 0.80 value of power = 0.84; s_1_ = standard deviation of the outcome variable of group 1 = 0.05; s_2_ = standard deviation of the outcome variable of group 2 = 0.07; M_1_ = mean of the outcome variable of group 1 = 0.42; M_2_ = mean of the outcome variable of group 2 = 0.26. The predicted sample size (n) for IA% measurements was found to be a total of 3.7 samples per group. In order to enhance the power of our results, a sample size of 10 subjects per group was determined for the IA% and CP measurements.

The statistical analyses were conducted using the SPSS software program (Version 28.0; IBM, Armonk, NY, USA). The Kolmogorov–Smirnov test was employed to ascertain the normality of the data distribution. This was followed by the implementation of a parametric statistical test. The internal gap volume-to-total interface volume ratio, along with the CP volume of the samples, was subjected to comparison with the outcomes of the one-way analysis of variance (ANOVA). Tukey’s post hoc adjustment was utilized for the purpose of conducting multiple comparisons. Univariate analysis of variance was applied to test the effect size of the filling method (layered vs. flow + layered vs. bulk), adhesive (self-etch vs. total-etch), and their interaction on the IA%. *p* values below 0.05 were considered statistically significant.

## 3. Results

### 3.1. Micro-Computed Tomography Measurement—3D Internal Adaptation

**Figure 3:**
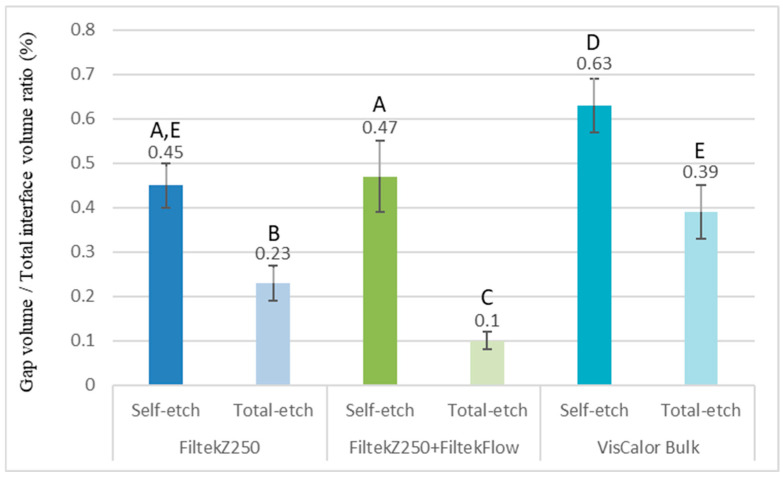
The ratio of interfacial gap volume to the total volume of the region of interest (ROI, designated examined cavity-restoration interfacial area), assessed using micro-computed tomography analysis. The utilization of different capital letters is indicative of a statistically significant difference, as determined using the one-way ANOVA and Tukey’s post hoc tests.

As demonstrated in Figure 3, the calculated ratio of interfacial gap volume to total interface volume is employed to analyze the IA.

A multiple comparison of the investigated groups in terms of the internal gap ratio is presented in Table 3. The most substantial gap formation exhibiting a significant discrepancy from the other groups (*p* < 0.001) was identified in Group 3/A, which underwent restoration with preheated VisCalor Bulk in conjunction with universal adhesive in self-etch mode (internal gap ratio: 0.63%). It was observed that Group 1/B, which involved the restoration of the cavity with a layered conventional RBC and an additional lining with flowable RBC, yielded the most favorable outcomes (internal gap ratio: 0.10%). With regard to the choice of adhesive system, the universal adhesive in the self-etch mode yielded results that were significantly less optimal than those of the groups treated with two-step total-etch adhesive (*p* < 0.001; 95% CI: 0.23–0.32). The application of one-way analysis of variance (ANOVA) to stratified samples in relation to adhesives revealed significant variations among all filling techniques in total-etch mode (*p* < 0.001). In contrast, VisCalor demonstrated a substantial reduction in IA in the self-etch mode [FZF_SE vs. VC_SE *p* < 0.001, 95% CI: −0.22 − (−0.08); FZ_SE vs. VC_SE *p* < 0.001, 95% CI: −0.24 − (−0.11)]. This finding was in contrast to the layered techniques, where the discrepancy was not deemed to be significant (FZF_SE vs. FZ_SE *p* = 0.72, 95% CI: −0.05–0.09).

Univariate analysis of variance demonstrated a significant effect of the *filling method* and the *adhesive type* on the IA (F(2,54) = 95.46, *p* < 0.001; F(1,54) = 399,19, *p* < 0.001, respectively). The partial eta-squared was considered to be large (ƞp^2^ = 0.78 and 0.88, respectively). Their interaction (*filling method* × *adhesive type*) was also found to be a significant contributing factor to the gap formation [F(5,54) = 11.69, *p* = < 0.001; ƞp^2^ = 0.30]. The observed power for the *filling method* and *adhesive type* was 0.96 and 1.00, respectively.

**Figure 4:**
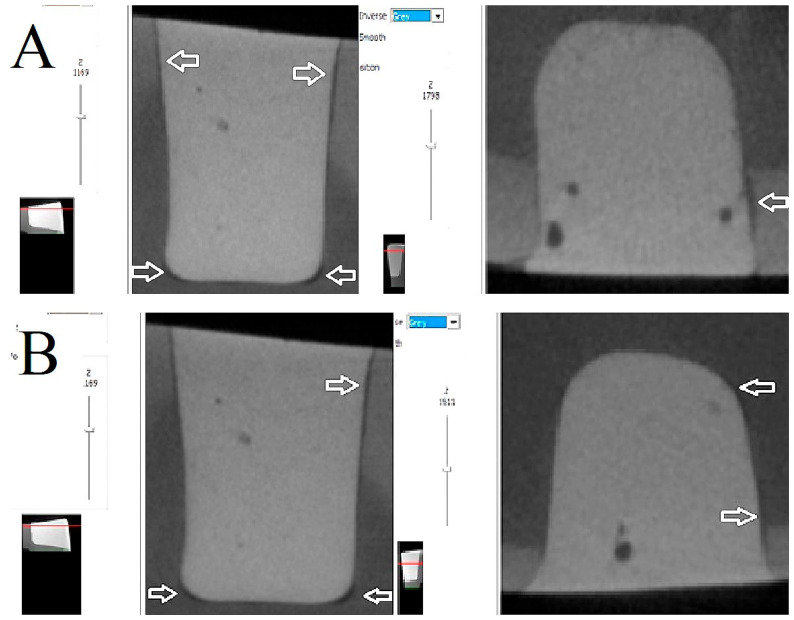
Representative images of microcomputed tomography analysis of Filtek Z250 layered restorations bonded with self-etch (**A**) and total-etch (**B**) adhesives. The presence of white arrows denotes the location of the internal gap.

**Figure 5:**
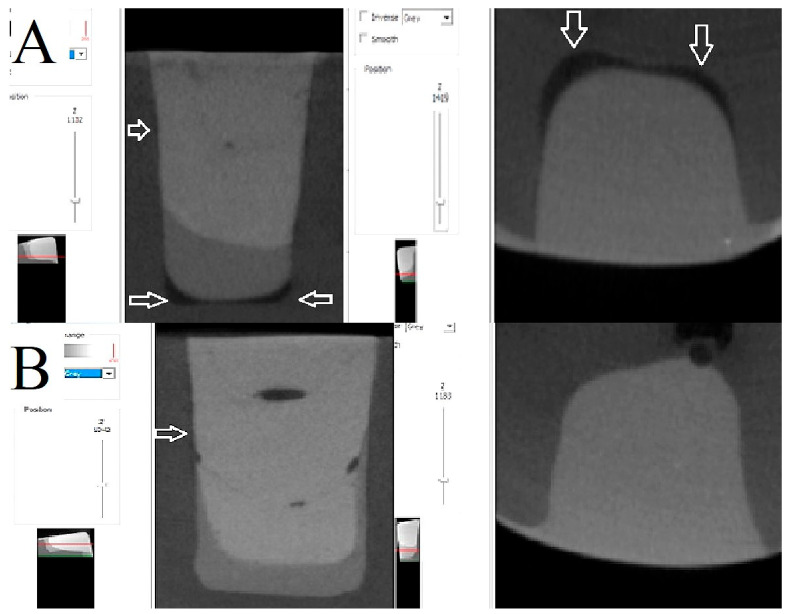
Representative images of microcomputed tomography analysis of Filtek Z250 layered restorations in combination with Filtek Supreme Flowable base, bonded with self-etch (**A**) and total-etch (**B**) adhesives. The presence of white arrows denotes the location of the internal gap.

**Figure 6:**
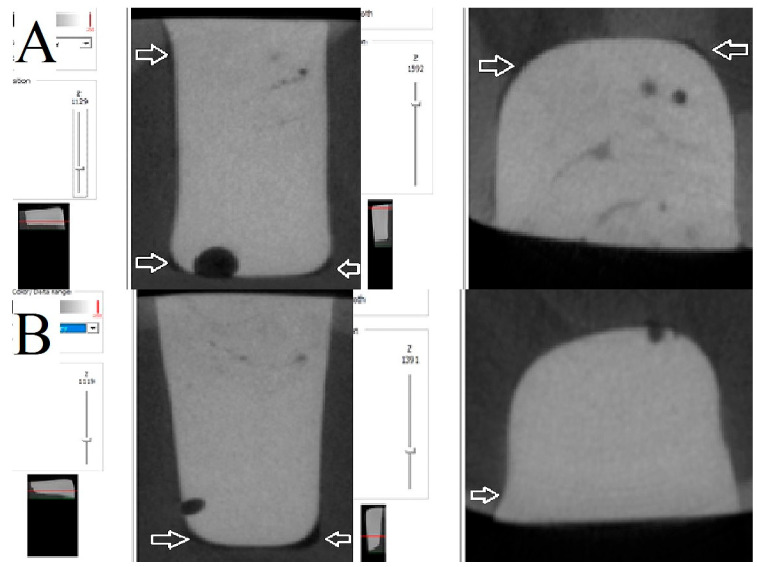
Representative images of microcomputed tomography analysis of preheated VisCalor Bulk restorations bonded with self-etch (**A**) and total-etch (**B**) adhesives. The presence of white arrows denotes the location of the internal gap.

As illustrated in Figure 4, Figure 5 and Figure 6, the images depict the IA and CP of the Filtek Z250 layered restoration, with and without the Filtek Supreme Flowable Base, and the preheated VisCalor Bulk restoration in combination with self-etch or total-etch adhesives.

With regard to the localization of the internal gaps formed, the majority of debonding was found on the lateral wall of the cavities. In the case of self-etch adhesives, this was often formed in the enamel–dentin junction area, involving the enamel.

### 3.2. Micro-Computed Tomography Measurement—3D Closed Porosity

**Figure 7:**
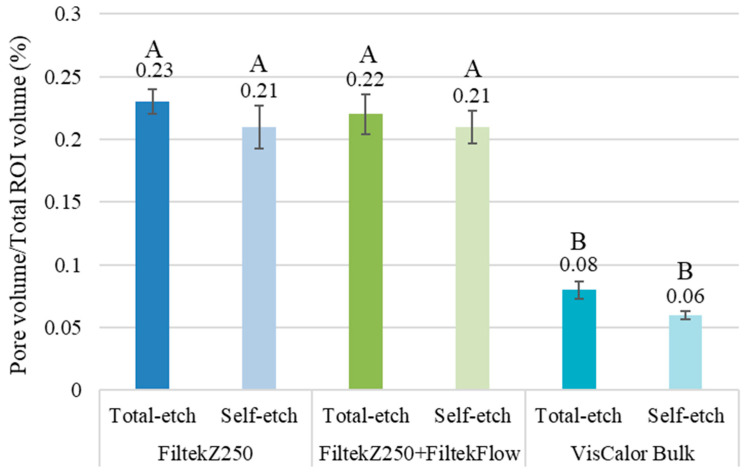
The ratio of closed pore volume to the total volume of the region of interest (ROI, resin composite restoration) was evaluated using micro-computed tomography analysis. The utilization of different capital letters is indicative of a statistically significant difference, as determined by the one-way ANOVA and Tukey’s post hoc tests.

As illustrated in Table 4, a multiple comparison of the groups under investigation has been conducted with regard to the CP volume in relation to the total volume of the restoration. The three-dimensional evaluation demonstrated that the preheated VisCalor Bulk exhibited significantly diminished values with respect to CP, when considered as a proportion of the total volume of the RBC sample (*p* = 0.006, 95% CI: 0.04–0.24) (Figure 7). No significant differences were identified between the samples prepared using the simple layering technique or the layering technique in combination with a flowable RBC base (*p* = 1.000, 95% CI: −0.10–0.10). The adhesive technique used had no discernible effect on CP (*p* = 0.518, 95% CI: −0.09–0.05).

Univariate analysis of variance demonstrated a significant effect of the *filling method* [F(2,54) = 18.01, *p* < 0.001], yet no significant effect of the *adhesive type* was observed with regard to the IA [F(1,54) = 0.97, *p* = 0.33]. The partial eta-squared was considered to be large for the *filling method* (ƞp^2^ = 0.40) and small the *adhesive type* (ƞp^2^ = 0.02). Their interaction (*filling method* * *adhesive type*) had a negligible effect on the CP [F(2,54) = 0.18, *p* = 0.84; ƞp^2^ = 0.01]. The observed power for the *filling method* and *adhesive type* was 1.00 and 0.16, respectively.

## 4. Discussion

The objective of this ex vivo study was to evaluate the IA and porosity of different restorative strategies employed in Class II cavities, using micro-CT analysis. The materials that were the focus of the investigation comprised thermoviscous bulk-fill RBC (VisCalor Bulk), a conventional RBC (Filtek Z250) applied in 2 mm increments with and without a flowable RBC liner (Filtek Supreme Flow), in combination with either a two-step total-etch or a universal self-etch adhesive. The findings of this study demonstrated that both the type of restorative technique and the adhesive strategy employed had a significant impact on the quality of internal adaptation. The highest volume of interfacial gaps was observed in the VisCalor Bulk group when used with the universal self-etch adhesive. In contrast, the most optimal adaptation was achieved through the combination of the stratification technique and the flowable RBC liner using a two-step total-etch adhesive system. Consequently, the null hypothesis—that there was no difference in IA among the tested RBCs—was rejected. The second null hypothesis, which stated that there is no difference in IA between the adhesive systems utilized, should also be rejected. This outcome can be attributed to the statistical analysis, which revealed substantial disparities between adhesives in favor of the two-step total-etch adhesive. The investigation revealed that the application of the layering technique resulted in a significantly higher number of voids in the restoration compared to the bulk-fill RBC restoration, as measured using micro-CT. Consequently, the third null hypothesis was refuted, which postulated that there was no difference in closed porosity among the tested RBCs.

The inferior performance of the VisCalor Bulk in combination with the universal adhesive in self-etch mode may be attributed to several factors. Initially, preheating the RBC improves flowability and adaptation; however, it may also accelerate polymerization kinetics and, in theory, may increase shrinkage stress once light curing begins [20,23,39]. A comparison of BisGMA-containing (VisCalor) and BisGMA-free RBCs revealed that the shrinkage strain was significantly higher for preheated BisGMA-containing RBCs [30]. In contrast, other research has revealed that preheating RBCs does not lead to an increase in shrinkage strain for some types of RBCs, due to a reduction in their viscosity [40,41]. A number of studies have indicated that VisCalor Bulk exhibits a reduced level of shrinkage stress in comparison to other bulk RBCs [31,32].

The viscoelastic properties of RBCs are known to be temperature-dependent. It is evident that an increase in temperature will result in an increase in energy, and consequently, the velocity of the particles will also increase. It has been demonstrated that achieving a pre-polymerization temperature of 45–60 °C results in a viscosity reduction of at least 30–84% [42,43]. However, the material cools immediately upon removal from the heating apparatus. This alteration transpires within a brief interval. It was ascertained that 50% of the attained temperature was forfeited within 120 s, and approximately 90% within 300 s [44]. An experimental investigation was conducted for the purpose of ascertaining the precise temperature change of VisCalor heated in a dispenser. The research test yielded a measurement of 60 °C, a result that was significantly lower than the stipulated 68 °C. A temperature drop of 26 °C was demonstrated from the time of heating until the initiation of polymerization during the application of the RBC [45]. Following this process, the viscoelastic properties of RBCs undergo a rapid transition. As the temperature is reduced, there is a significant increase in viscosity, which leads to a reduction in flowability and an impaired capacity to adapt to the internal contours of the cavity. Furthermore, as the material cools, its elastic modulus increases, resulting in a stiffer consistency that is less capable of compensating for polymerization shrinkage stress. However, it has been demonstrated that the elevated pre-polymerization temperature, in conjunction with the heat generated during polymerization, can substantially augment the thermal expansion coefficient mismatch between the polymer matrix and the fillers. This can result in the development of stress at the filler–matrix interface and the generation of internal stresses around the fillers [46]. As demonstrated in [47], there appears to be a direct proportionality between residual stress and temperature increase. Furthermore, the thermal volumetric change exhibited by RBCs is found to be six to eight times greater than that of the surrounding tooth structures [22]. Polymerization shrinkage in conjunction with thermal contraction has been shown to generate elevated interfacial stresses in preheated RBCs upon thermal equilibrium, exerting deleterious effects on internal and marginal adaptation [48]. Additionally, the kinetics of polymerization can be influenced by the temperature of the material during the curing process. It has been hypothesized that RBCs with higher temperatures may demonstrate accelerated polymerization, a reduced gel phase, and an augmented degree of conversion. This may result in a diminished capacity to alleviate stress through flow during the curing process [49]. Consequently, the benefits of preheating are highly time-sensitive. It is important to note that any delay in placement may negate these advantages [27], potentially resulting in poorer adaptation than expected.

The adhesive system employed, or indeed the combination of adhesive system and RBC type, also played a crucial role in marginal sealing. A comparison of adhesive systems across all restorative techniques was conducted, and the results demonstrated that the self-etch application mode of the universal adhesive resulted in significantly higher gap formation than the two-step total-etch approach. This outcome is consistent with the results of other in vitro studies that evaluated the microleakage of Class II restorations using the same self-etch adhesive, which was employed in the present study. The total-etch and selective etch techniques have been demonstrated to exhibit superior performance in comparison to self-etch adhesive [50,51]. Etching dentin with phosphoric acid effectively removes the smear layer while exposing the collagen matrix, thus enabling better infiltration of the adhesive and stronger hybrid layer formation [52]. Despite the evident advantages offered by universal adhesives in terms of ease of application, it is important to note that their performance is subject to significant variation depending on the composition of the adhesive and the method of application [53]. Notwithstanding the presence of 10-methacryloyloxydecyl dihydrogen phosphate (10-MDP) and pentaerythritol (PENTA) monomers, it was demonstrated that the 2-hydroxyethyl methacrylate (HEMA)-free Prime & Bond Universal exhibited the weakest bond strength in self-etch mode when compared to other universal adhesives utilizing divergent etching strategies [54]. It has been demonstrated that 10-MDP enhances adhesion by forming a stable ionic bond with dentin calcium [55]. However, the erythritol phosphate group of the polymer network strengthener PENTA may impede calcium bonding due to steric hindrance [54]. The substandard performance observed in the self-etch mode can be attributed to the use of the solvent isopropanol, which possesses a lower dielectric constant compared to ethanol. It has been hypothesized that this may result in an increase in the pKa of acidic monomers, leading to a reduction in hydrolyzed species and an impairment of calcium interaction [56]. Furthermore, in comparison to ethanol, the weak hydrogen bonding capacity of isopropanol can render the adhesive less effective at breaking interpeptide hydrogen bonds that would stabilize collagen fibrils. The lower stiffening rate of the former may increase matrix shrinkage and reduce resin infiltration [57]. In addition, research has demonstrated that the pH of self-etch adhesives can significantly impact the strength of the adhesive bond [58]. The employment of ultra-mild (pH > 2.5) or mild (pH 2–2.5) universal adhesives is associated with limitations in terms of their capacity to penetrate the smear layer. This phenomenon has the potential to compromise the short- and long-term bonding stability [54]. A comprehensive meta-analysis incorporating both in vivo and in vitro studies also confirms that the formation of internal and marginal gaps varies depending on the type of adhesive used. In the majority of cases, etch-and-rinse adhesives demonstrated superior performance, irrespective of the RBC filling application technique employed [59]. This outcome lends support to the hypothesis that, while universal adhesives offer versatility, their bonding efficacy—particularly in self-etch mode—may be inadequate in high C-factor cavities or in enamel-rich regions, unless additional selective enamel etching is employed [60,61,62].

In addition to the aforementioned issues, it has been established that the bulk utilization of RBC is known to engender augmented polymerization shrinkage stress due to the increased volume of the cured material. This phenomenon may contribute to the formation of gaps if adequate compensation is not provided by the adhesive interface [63]. Conversely, a meta-analysis study has demonstrated that bulk-fill RBCs exhibit superior polymerization shrinkage and shrinkage stress values in comparison to conventional RBCs [64]. In the present study, it was demonstrated that restorations placed incrementally with a conventional RBC, especially those with a flowable liner, exhibited superior adaptation. The utilization of a flowable RBC as a stress-absorbing intermediate layer is hypothesized to have enhanced marginal seal by compensating for polymerization shrinkage and adapting more effectively to the cavity walls. These findings are consistent with those of previous studies, which reported enhanced IA with flowable liners and incremental layering techniques in comparison to bulk-fill strategies [65,66,67]. In contrast, other studies have demonstrated that a flowable liner is less effective than preheating the RBC in reducing microleakage [27]. Furthermore, research has indicated that the utilization of preheated VisCalor in lieu of non-preheated bulk-fills or layered conventional RBC can result in a reduction in the formation of marginal or internal gaps [32,33,68].

A further salient point pertains to the interpretation of the results: given the necessity to standardize the samples for an in vitro study, the cavities were prepared with blue diamond burs (~100 µm grit size). It is hypothesized that this process resulted in the formation of a thick and dense smear layer on the surface of the dentin. It has been demonstrated through rigorous research that the smear layer left behind by the diamond abrasive is challenging to penetrate, particularly for mild and ultra-mild self-etching systems. This has been shown to result in impaired bond strength [69]. The detrimental effect of the unpenetrated smear layer can further exacerbate the formation of unfavorable shrinkage vectors caused by a high C-factor cavity, which already has a deleterious effect on adhesion [70]. Notwithstanding the advantages of bulk-fill restoratives, including ease of application and reduced chair time, it has been demonstrated that not all bulk fills can achieve the same level of adhesion as conventional layered materials in high C-factor cavities. In addition, the etch-and-rinse approach has been shown to result in enhanced bond strength and superior resistance to the deleterious effects of ageing in cases where high C-factor cavities were restored with bulk-fill RBCs [71].

While the internal adaptation of VisCalor Bulk with both self-etch and total-etch adhesive systems demonstrated inferior results in comparison to those achieved with conventional RBCs, significantly superior outcomes were observed in relation to closed porosity. It is important to note that internal pores and voids in RBC restorations can be regarded as defects. Such defects have been shown to have a detrimental effect on the material’s strength, thus increasing the risk of fracture. Furthermore, these defects can lead to microleakage, which can potentially result in secondary caries [72]. The findings of this study are consistent with the extant literature, which demonstrates that VisCalor Bulk exhibited the lowest void ratio subsequent to the adaptation of bulk-fill RBCs to the cavity, as determined using micro-CT analysis [73,74]. In contrast, a study that evaluated the effect of preheating on the internal pore formation of high-viscosity conventional layered and bulk-filled samples found that elevated pre-polymerization temperatures significantly increased the closed pore volume relative to the total volume of RBC samples [35]. In essence, void formation is a multifactorial phenomenon influenced by a number of factors. The factors to be considered include the polymerization kinetics of the material, the composition of the resin matrix, the filler load, the architecture of the polymer network, and its heterogeneity [75]. Nevertheless, the presence of fewer internal voids is observed in more flowable materials and when the application of heat is employed [76]. However, a recent study has demonstrated that preheating alone does not reduce the formation of internal voids in RBCs, including VisCalor Bulk. Rather, injection has been shown to have a beneficial effect [34].

However, it is imperative to acknowledge the limitations of the study, which are as follows:

Notwithstanding the samples having been immersed in distilled water for a period of three months following the restorative procedure, this does not take into account the intraoral variables such as thermal cycling, occlusal loading, and long-term hydrolytic degradation. As demonstrated in [59], these factors have been shown to be detrimental to marginal adaptation, irrespective of the material that is applied and the technique by which it is applied. Consequently, in order to obtain results that are more closely aligned with the clinical situation, it is proposed that similar studies in the future be complemented with thermocycling, aging, and mechanical loading simulating chewing. In the present study, a limited number of RBC-adhesive combinations were examined. Consequently, it is not possible to extrapolate these findings to all cases. A comprehensive investigation into the properties of various RBCs and adhesive combinations is warranted as a future research direction, with the aim of identifying the most efficacious combinations for the purpose of minimizing internal gap formation and porosity. It can be hypothesized that, due to the reduced distance, there will be an increased degree of conversion and possibly increased polymerization shrinkage. Despite the minor discrepancies between this experimental protocol and the actual clinical scenario, all samples were subjected to the same protocol. This ensures a valid basis for comparative analysis of the restorative procedures.

Furthermore, although micro-CT provides non-destructive 3D evaluation, its resolution may limit the detection of micro-gaps smaller than the voxel size. Moreover, earlier studies have indicated that high-dose X-ray exposure, particularly in the context of small samples and polymer-based materials, has the potential to induce structural alterations or polymer degradation during micro-CT scanning [77]. In the context of RBCs, it has been demonstrated that the presence of a high ratio of inorganic filler content relative to the organic matrix can result in the attenuation of ionizing radiation. Consequently, no discernible mechanical or thermal change is identifiable [77]. In order to minimize potential radiation-induced artefacts, the scan parameters (tube voltage, current, and exposure time) were meticulously optimized to achieve a balance between image quality and sample integrity. Nevertheless, micro-CT remains the gold standard for highly accurate imaging in experimental dental research due to its ability to deliver reproducible, high-resolution visualizations and quantitative analyses of internal adaptation, polymerization shrinkage, mineral density, and material porosity, among others. The technology under discussion facilitates the extraction of detailed 2D and 3D data of mineralized hard tissues, soft tissues, as well as solid and liquid materials, that would otherwise be unattainable using conventional techniques [78,79,80].

Future research should be expanded to encompass mechanical testing, including microtensile bond strength, and the implementation of ageing protocols. These additional studies will facilitate enhanced prediction of the restorations’ behaviour and the potential clinical durability

## 5. Conclusions

The findings of this ex vivo study suggest that thermoviscous VisCalor Bulk RBC may offer advantages in reducing internal porosity. However, its internal adaptation was found to be inferior, particularly when combined with Prime & Bond Universal adhesive in a self-etch strategy. In contrast, incremental layering with conventional Filtek Z250 RBC, especially when used with a flowable liner (Filtek Supreme Flowable Restorative) and a total-etch adhesive (Adper Single Bond 2), proved effective in minimizing interfacial gaps. This technique, though, has been observed to be associated with an increase in internal porosity.

## Figures and Tables

**Table 1:** Materials, manufacturers, and composition of the investigated resin composites.

Type of the Material	Brand Name	Manufacturer	Matrix	Filler	Filler Load
Conventional high-viscosity resin composite	Filtek Z250	3M ESPE, St. Paul, MN, USA	BisGMA, BisEMA, TEGDMA, UDMA	0.01–3.5 µm (mean 0.6) Zr-silica	80 wt%
Conventional low-viscosity resin composite	Filtek Supreme Flowable Restorative	3M ESPE, St. Paul, MN, USA	BisGMA, TEGDMA, Procrylat resin	20–75 nm silica, 0.6–10 µm cluster Zr-silica, 0.1–5 µm YbF3	65 wt%
Thermoviscous bulk-fill resin composite	VisCalor Bulk	Voco, Cuxhaven, Germany	BisGMA, aliphatic DMA	nano-hybrid (not detailed by the company)	83 wt%
One-step self-etch universal dental adhesive	Prime & Bond Universal	Dentsply Sirona, Charlotte, NC, USA	PENTA, 10-MDP, Active Guard ^TM^ crosslinker, isopropanol, water	unfilled	
Two-step total-etch dental adhesive	Adper Single Bond 2	3M ESPE, St. Paul, MN, USA	BisGMA, HEMA, dimethacrylates, ethanol, water, polyacrylic, polyitaconic acids	5 nm silica	10 wt%

Abbreviations: BisGMA: bisphenol-A diglycidyl ether dimethacrylate; BisEMA: bisphenol-A polyethylene glycol diether dimethacrylate; DMA: dimethacrylate; HEMA: hydroxyethyl methacrylate; PENTA: dipentaerythritol pentacrylate phosphate; TEGDMA: triethylene glycol dimethacrylate; UDMA: urethane dimethacrylate; 10-MDP: 10-methacryloyloxydecyl dihydrogen phosphate; wt%: weight%.

**Table 2:** Study group arrangements.

Group	Subgroup	Adhesive	Materials	Group Code	Sample Size
Group 1.	A	Prime & Bond Universal	2 mm thick oblique layers of Filtek Z250	FZ_SE	n = 10
	B	Adper Single Bond 2		FZ_TE	n = 10
Group 2.	A	Prime & Bond Universal	1 mm thick Filtek Supreme Flow base at the gingival floor + 1.5 mm thick oblique layers of Filtek Z250	FZF_SE	n = 10
	B	Adper Single Bond 2		FZF_TE	n = 10
Group 3.	A	Prime & Bond Universal	4 mm thick bulk-filled preheated (68 °C) VisCalor Bulk	VC_SE	n = 10
	B	Adper Single Bond 2		VC_TE	n = 10

Abbreviation: SE, self-etch adhesive (Prime & Bond Universal); TE, total-etch (Adper Single Bond 2).

**Table 3:** Multiple comparison of the investigated groups in terms of the internal gap ratio (one-way ANOVA and Tukey’s post-hoc test).

Comparison	Mean Difference	S.D.	*p* Value	95% Confidence Interval	
				Lower	Upper
FZ_SE vs. FZF_SE	−0.02	0.02	0.94	−0.09	0.05
FZ_SE vs. FZF_TE	0.35	0.02	<0.01	−0.28	0.42
FZ_SE vs. FZ_TE	0.22	0.02	<0.01	0.15	0.29
FZ_SE vs. VC_SE	−0.18	0.03	<0.01	−0.25	−0.11
FZ_SE vs. VC_TE	0.06	0.01	0.18	−0.01	0.13
FZ_TE vs. FZF_SE	−0.25	0.02	<0.01	−0.32	−0.17
FZ_TE vs. FZF_TE	0.12	0.03	<0.01	0.05	0.19
FZ_TE vs. VC_SE	−0.40	0.02	<0.01	−0.45	−0.33
FZ_TE vs. VC_TE	−0.17	0.02	<0.01	−0.24	−0.09
FZF_SE vs. FZF_TE	0.37	0.03	<0.01	0.29	0.44
FZF_SE vs. VC_SE	−0.15	0.02	<0.01	−0.22	−0.08
FZF_SE vs. VC_TE	0.08	0.02	0.02	0.01	0.15
FZF_TE vs. VC_SE	−0.52	0.03	<0.01	−0.59	−0.45
FZF_TE vs. VC_TE	−0.29	0.02	<0.01	−0.36	−0.22
VC_SE vs. VC_TE	−0.23	0.03	<0.01	−0.31	−0.16

Abbreviations: SE, self-etch; TE, total-etch; FZ, FiltekZ250 layered; FZF, FiltekZ250 layered and Filtek Supreme Flowable base; VC, preheated VisCalor Bulk.

**Table 4:** Multiple comparison of the test groups for closed porosity (one-way ANOVA and Tukey’s post-hoc test).

Comparison	Mean Difference	S.D.	*p* Value	95% Confidence Interval	
				Lower	Upper
FZ_SE vs. FZF_SE	−0.01	0.04	1	−0.12	0.11
FZ_SE vs. FZF_TE	−0.01	0.04	0.99	−0.13	0.1
FZ_SE vs. FZ_TE	−0.02	0.04	0.99	−0.13	0.09
FZ_SE vs. VC_SE	0.15	0.04	<0.01	0.04	0.26
FZ_SE vs. VC_TE	0.11	0.02	0.06	−0.01	0.22
FZ_TE vs. FZF_SE	0.01	0.04	0.99	−0.09	−0.17
FZ_TE vs. FZF_TE	0.01	0.03	1	−0.01	0.12
FZ_TE vs. VC_SE	0.17	0.04	<0.01	0.06	0.28
FZ_TE vs. VC_TE	0.13	0.04	0.01	0.02	0.25
FZF_SE vs. FZF_TE	−0.01	0.04	1	−0.12	0.11
FZF_SE vs. VC_SE	0.16	0.04	<0.01	0.04	0.27
FZF_SE vs. VC_TE	0.12	0.04	0.03	0.01	0.23
FZF_TE vs. VC_SE	0.16	0.04	<0.01	0.05	0.28
FZF_TE vs. VC_TE	0.12	0.04	0.02	0.01	0.24
VC_SE vs. VC_TE	−0.04	0.03	0.92	−0.08	0.15

Abbreviations: SE, self-etch; TE, total-etch; FZ, FiltekZ250 layered; FZF, FiltekZ250 layered and Filtek Supreme Flowable base; VC, preheated VisCalor Bulk.

## Data Availability

The datasets generated during and/or analyzed during the current study are available from the corresponding author upon reasonable request.

## Abbreviations

The following abbreviations are used in this manuscript:

RBC	Resin-based composite
IA	Internal adaptation
CP	Closed porosity
Micro-CT	Micro-computed tomography
BisGMA	Bisphenol-A diglycidyl ether dimethacrylate
BisEMA	Bisphenol-A polyethylene glycol diether dimethacrylate
DMA	Dimethacrylate
HEMA	Hydroxyethyl methacrylate
PENTA	Dipentaerythritol pentacrylate phosphate
TEGDMA	Triethylene glycol dimethacrylate
UDMA	Urethane dimethacrylate
10-MDP	10-methacryloyloxydecyl dihydrogen phosphate
wt%	Weight%
SE	Self-etch adhesive
TE	Total-etch adhesive
FZ_SE	Filtek Z250 layered_self-etch adhesive
FZ_TE	Filtek Z250 layered_total-etch adhesive
FZF_SE	Filtek Z250 layered + Filtek Supreme Flowable base_self-etch adhesive
FZF_TE	Filtek Z250 layered + Filtek Supreme Flowable base_total-etch adhesive
VC_SE	Preheated VisCalor Bulk_self-etch adhesive
VC_TE	Preheated VisCalor Bulk_total-etch adhesive
ROI	Region of interest
ANOVA	One-way analysis of variance
CI	Confidence interval
